# Low-Abundance HIV Drug-Resistant Viral Variants in Treatment-Experienced Persons Correlate with Historical Antiretroviral Use

**DOI:** 10.1371/journal.pone.0006079

**Published:** 2009-06-29

**Authors:** Thuy Le, Jennifer Chiarella, Birgitte B. Simen, Bozena Hanczaruk, Michael Egholm, Marie L. Landry, Kevin Dieckhaus, Marc I. Rosen, Michael J. Kozal

**Affiliations:** 1 Yale University School of Medicine, New Haven, Connecticut, United States of America; 2 454 Life Sciences/Roche, Branford, Connecticut, United States of America; 3 University of Connecticut, Storrs, Connecticut, United States of America; 4 VA Connecticut Healthcare System, New Haven, Connecticut, United States of America; University of Toronto, Canada

## Abstract

**Background:**

It is largely unknown how frequently low-abundance HIV drug-resistant variants at levels under limit of detection of conventional genotyping (<20% of quasi-species) are present in antiretroviral-experienced persons experiencing virologic failure. Further, the clinical implications of low-abundance drug-resistant variants at time of virologic failure are unknown.

**Methodology/Principal Findings:**

Plasma samples from 22 antiretroviral-experienced subjects collected at time of virologic failure (viral load 1380 to 304,000 copies/mL) were obtained from a specimen bank (from 2004–2007). The prevalence and profile of drug-resistant mutations were determined using Sanger sequencing and ultra-deep pyrosequencing. Genotypes were interpreted using Stanford HIV database algorithm. Antiretroviral treatment histories were obtained by chart review and correlated with drug-resistant mutations. Low-abundance drug-resistant mutations were detected in all 22 subjects by deep sequencing and only in 3 subjects by Sanger sequencing. In total they accounted for 90 of 247 mutations (36%) detected by deep sequencing; the majority of these (95%) were not detected by standard genotyping. A mean of 4 additional mutations per subject were detected by deep sequencing (p<0.0001, 95%CI: 2.85–5.53). The additional low-abundance drug-resistant mutations increased a subject's genotypic resistance to one or more antiretrovirals in 17 of 22 subjects (77%). When correlated with subjects' antiretroviral treatment histories, the additional low-abundance drug-resistant mutations correlated with the failing antiretroviral drugs in 21% subjects and correlated with historical antiretroviral use in 79% subjects (OR, 13.73; 95% CI, 2.5–74.3, p = 0.0016).

**Conclusions/Significance:**

Low-abundance HIV drug-resistant mutations in antiretroviral-experienced subjects at time of virologic failure can increase a subject's overall burden of resistance, yet commonly go unrecognized by conventional genotyping. The majority of unrecognized resistant mutations correlate with historical antiretroviral use. Ultra-deep sequencing can provide important historical resistance information for clinicians when planning subsequent antiretroviral regimens for highly treatment-experienced patients, particularly when their prior treatment histories and longitudinal genotypes are not available.

## Introduction

HIV genotyping technologies other than the conventional HIV genotyping have been used to show that viral variants in an HIV-infected person, whether acutely or chronically infected, are more genetically diverse than previously appreciated by conventional HIV genotyping assays [1]–[9]. Current genotyping assays are based on population sequencing of reverse transcriptase - polymerase chain reaction (RT-PCR) generated products of HIV protease (PR) and reverse transcriptase (RT) genes. Although this technology has been a major advancement in the understanding and management of HIV drug resistance in clinical practice, a major limitation is the inability to detect low-abundance drug-resistant mutations (DRMs) at levels <20% of the viral quasi-species [10], [11]. Low-abundance drug-resistant HIV variants can occur de novo through the extraordinary HIV genetic diversity generated via highly error-prone replication [12] or as the result of transmitted resistant strains that persist within an infected individual [6], [7], [13]. Understanding the environments in which low-abundance drug-resistant variants develop, how they evolve and impact treatment response are important areas that require further investigations.

A growing number of studies have shown that low-abundance DRMs can be detected in chronically-infected antiretroviral-naïve individuals using ultra-sensitive allele-specific PCR assays or by ultra-deep sequencing methods [8], [14]–[17]. These studies show that baseline low-abundance DRMs undetected by conventional sequencing, in particular non-nucleoside reverse transcriptase inhibitor (NNRTI) mutations, are associated with poor treatment response in persons initiating antiretroviral therapy (ART). This finding follows the Darwinian principle of ‘survival of the fittest’, in that drug-resistant variants at low levels can out-compete wild-type virus in presence of antiretroviral selection pressure and lead to treatment failure. A common and clinically-relevant question that clinicians ask is how often commercial HIV genotyping underestimates the presence of low-abundance DRMs in treatment-experienced patients being evaluated for virologic failure, and whether unrecognized low-abundance DRMs can contribute to virologic failure. In this study, we examine the prevalence and patterns of low-abundance DRMs in antiretroviral-experienced subjects experiencing virologic failure using standard Sanger sequencing and a new ultra-deep sequencing method [9], [16], [18]. We characterize how the addition of low-level DRMs affects the resistance burden in antiretroviral-experienced subjects using the Stanford HIV drug resistance database algorithm and describe the relationship of the low-abundance DRMs to subjects' failing antiretroviral drugs and their longitudinal antiretroviral histories.

## Materials and Methods

### Study sample selection

Plasma samples from 22 different HIV-infected antiretroviral-experienced subjects who had HIV genotype testing done for virologic failure indication (viral loads ranging from 1380 to 304,000 copies/mL) were obtained from a specimen bank collected between 2004–2007 from routine HIV care at Yale New Haven Hospital and from an adherence-focused clinical trial in Connecticut [19]. All samples were identified only by a lab number during standard and ultra-deep sequencing analyses. Once blinded drug resistance analyses were completed, chart reviews of patients' existing clinical information were performed, and subjects' antiretroviral treatment histories were merged with resistance data. Antiretroviral histories from clinical trial subjects (N = 12) were obtained from the study's existing electronic database. The study met criteria for informed consent exemption and was approved by Yale University School of Medicine and University of Connecticut human investigation committees.

### Standard population genotyping analysis

Viral RNA was isolated from 140 µl of plasma samples using QIAmp RNA Mini kit (QIAGEN, Valencia, CA). The extracted RNA specimens were subjected to RT-PCR using SuperScript III (Invitrogen, Carlsbad, CA) to make cDNAs. A PCR assay previously described was used to amplify PR and RT genes [20], [21]. PCR amplicons were sequenced directly by population sequencing using an ABI 3730 XL automated sequencing system (Applied Biosystems, Foster City, CA). All amino acid positions associated with antiretroviral resistance according to IAS-USA 2008 and Stanford HIV drug resistance database were evaluated [22]. Nucleotide mixtures at drug-resistant sites were called if a discriminate peak from baseline was observed in two independent reactions.

### Ultra-deep pyrosequencing analysis

The same volume of plasma (140 µl) and RNA extraction protocol used for standard sequencing was used for deep sequencing to allow for a direct comparison of the two methods. Input HIV RNA was not measured for standard sequencing nor ultra deep sequencing for this set of samples. It has been shown that the stochastic effects of sampling variation become low when the starting RNA copy number in a specimen is ≥1,000 (or ≥10,000 HIV RNA copies/mL for an unextracted sample considering a 10% RNA extraction efficiency) [9], [23]. Thus for samples with RNA copy numbers <10,000 (#3, 9, 11, 16, 18, and 22), ultra-deep sequencing identifies the proportion of sequenced PCR amplicons containing the mutation and may not represent the actual proportion of HIV variants in a plasma sample.

Three gene-specific overlapping cDNA per sample were generated and subjected to 40 cycles of PCR with FastStart HiFi polymerase (Roche, Indianapolis, IN) to make eight partly overlapping amplicons that cover the entire PR and the first 236 codons of RT genes. The amplicons were purified with AMPure magnetic beads (Agencourt, Beverly, MA) and quantitated by PicoGreen fluorescence (Invitrogen). After equimolar pooling of all 8 amplicons per sample, clonal amplification on beads was performed using reagents enabling sequencing in both forward (kit II) and reverse (kit III) directions (Roche/454 Life Sciences, Branford, CT). Kit II and III emulsions were pooled for each sample before bead isolation. After enrichment of DNA-containing beads, these were counted on a Multisizer3 Coulter counter.

Approximately thirty-thousand beads per sample were prepared for ultra-deep sequencing and loaded on a PicoTiter plate fitted with a 16-lane gasket. Sequencing was performed on a Genome Sequencer FLX (Roche/454 Life Sciences). An average of 1700 reads per nucleotide position was obtained for this set of samples, which allowed for accurate detection of variants down to approximately 1% when viral load is >10,000 copies/mL. Ultra-deep sequencing reproducibility and level of variant detection have been previously reported [9], [18]. The sensitivity of ultra-deep sequencing in its current platform for detection of low-level viral variants at levels 0.1 to 1% has been confirmed by used of standard cloning methods [9], [18], [24], [25]. In this study we reported a variant detection limit of ≥1% as resistant variants at this level have been shown to be clinically relevant [7], [14]–[16].

Amplicon Variant Analyzer software was used to align all amplicon sequence reads to a consensus sequence generated from over 6000 sequences found in the Los Alamos HIV sequence database for identification of all nucleotide changes of interest. Further detailed of this technology can be found elsewhere [9], [16], [18].

### Statistical analysis

**Figure 1:**
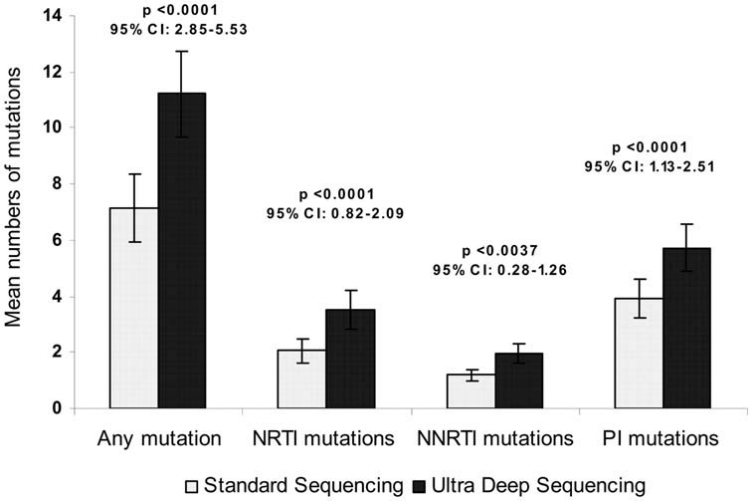
Mutation detection by standard versus ultra-deep sequencing according to mutation classes. A mean of 4 additional mutations per subject were detected by ultra-deep sequencing. The difference in the number of mutations detected by the two methods were statistically significant for NRTI (nucleotide/nucleoside reverse transcriptase inhibitor), NNRTI (non-nucleotide reverse transcriptase inhibitor), and PI (protease inhibitor) mutations. Two-tailed p values and 95% confidence intervals were calculated from the paired Student T test.

Paired Student T test was used to compare the mean number of DRMs detected by ultra-deep versus standard sequencing in 22 samples for all mutations, nucleoside reverse transcriptase inhibitor (NRTI) mutations, NNRTI mutations and protease inhibitor (PI) mutations, respectively. All p values presented were two tailed, and 95% confidence intervals were calculated and reported in figure 1. Odds ratios were used to describe the association of low-abundance DRMs and subjects' current failing antiretroviral versus historical antiretroviral drugs. Fisher exact test was used to determine the statistical significance of the association.

### Definition of low-abundance drug-resistant mutations

DRMs that are not detected by standard sequencing but detected using more sensitive technologies have been referred to as “minority” or “low-level” drug-resistant variants in the literature [1]–[9]. For the purpose of discussion in this paper, we define “low-abundance” DRMs as mutations detected at <20% of viral quasi-species and “high-abundance” DRMs as mutations detected at ≥20% of viral quasi-species by ultra-deep sequencing.

## Results

### Comparison of ultra-deep vs. standard population sequencing data

Low-abundance DRMs were detected in all 22 subjects by deep sequencing and were unrecognized in 19 subjects by standard sequencing. In total they accounted for 90 of the 247 mutations (36%) detected by deep sequencing; the majority of these (95%) were not detected by standard genotyping. A mean of 4 (ranging from 1 to 10) additional mutations per subject were detected by deep sequencing compared to standard sequencing (p<0.0001, 95%CI: 2.85–5.53). The differences in the numbers of DRMs detected by the two methods remained statistically significant when mutations were classified into NRTIs, NNRTIs, and PIs (Figure 1).

DRMs that were detected at levels <13% of viral quasi-species by deep sequencing were not detected by standard sequencing in this study. Ultra-deep sequencing identified all DRMs at levels ≥20% that were detected by standard sequencing. By comparison, standard sequencing detected only 152 of 157 (97%) DRMs at levels ≥20% that were detected by deep sequencing. The high-abundance mutations that were not detected by standard sequencing were at levels between 20 to 30.5%. In total, only 57% of mutations at levels between 20 and 30.5% were identified by standard sequencing in this study.

### Low-abundance drug-resistant mutation profiles

Of the total 90 additional low-abundance DRMs detected by ultra-deep sequencing in subjects, 62% were detected at levels 1 to 5%, and 38% were detected at levels 5 to 20% (Figure 1). Low-abundance DRMs from all three antiretroviral classes were detected. Eight of 22 subjects (36.5%) harbored low-abundance mutations that imparted resistance to a single antiretroviral class; 6 of 22 subjects (27%) harbored resistance to two antiretroviral classes, and 8 of 22 subjects (36.5%) harbored resistance to three antiretroviral classes.

Low-abundance PI mutations were most commonly seen, occurring in 18 of 22 subjects (82%); however the majority was “minor” IAS-USA PI mutations [23]. Low-abundance NRTI and NNRTI mutations were also frequently detected, occurring in 17 of 22 subjects (73%) and 10 of 22 subjects (45%), respectively. Table 1 lists all mutations detected in each subject by standard and deep sequencing. The most common low-abundance NRTI mutations detected were thymidine analog mutations (TAMs) M41L, K70R, L210W, T215F/Y or K219E/Q. At least one of these was detected in 11 of 22 subjects. T215F/Y was the most common low-abundance TAMs detected. T215Y and T215F have been shown to be associated with two distinct TAM pathways. T215Y is associated with M41L, L210W, and sometimes D67N (TAM-1 pathway), and T215F is associated with D67N, K70R, and K219Q (TAM-2 pathway) [26], [27]. T215Y was observed to cluster with mutations in TAM-1 pathway (subjects #5 & 16) and also with mutations in TAM-2 pathway (subjects #6 & 13). T215F was either detected alone (subjects #11 & 15) or together with mutations from both TAM-1 and TAM-2 pathways (subjects #1 & 3). Low-abundance M184V was detected in only one of 22 samples. Low-abundance NNRTI mutations were detected in 10 of 22 subjects (45%) by deep sequencing and in only 1 of 22 subjects by standard sequencing (subject #1 - K103N at 13.9%). The most common low-abundance NNRTI mutations were K103N, followed by Y181C, Y188C and G190A in this set of samples.

**Table 1:** HIV drug resistance mutation detection by standard sequencing (ss) and ultra-deep sequencing (uds).

Pt.	Viral Load	Failing ARV regimen (date)	Previous ARV exposure (date)	Protease (% abundance by uds)		Reverse Transcriptase (% abundance by uds)	
				Mutations detected by both ss and uds	Mutations detected by uds only	Mutations detected by both ss and uds	Mutations detected by uds only
1	135,000	TDF ABC 3TC TPV/r (8/07)	AZT 3TC DDI EFV LPV/r FPV/r NFV	L10F (66.1), I13V (91.4), L33F (92.3), I54V (87.4), Q58E (80.9), D60E (82.6), I62V (91.4), L63P (99.4), A71V (91.5), G73T (90.8), V82C (87.7), I84V (93), L90M (90.8)	*L10V (17.9)*, ***V32I (1.1)***, *A71I (1.7), G73S (2.4), I85V (2.6)*	M41L (85), D67N (87), L74V (46.5), **K103N (13.9)**, V118I (35.6), Y181C (37.7), M184V (73.8), G190A (52.2), L210W (73), T215Y (70.1)	*E44D (9.2)*, ***K70R (1.1)***, *L74I (2.1)*, ***T215F (1.1)***
2	101,000	TDF FTC EFV (8/07)	No prior therapy	L63P (100), V77I (99.8)		A62V (29.9), K65R (94.4), K103N (98.4), V108I (99.8), M184V (99.9), P225H (99.4)	*T69I (2.1)*
3	5,760	ABC 3TC ATV/r (10/07)	D4T DDI EFV LPV/r (3/04)	L10I (99.5) , I13V (100), L33F (100), N36L (100), K43T (97.6), M46L (99.3), G48V (45.8), I62V (99.9), I64V (99.9), T74P (99.7), V82A (99.8), I84V (99.6)		D67N (22.1), K103N (22.5), M184V (21.7), G190A (20.8), **L210W (14.6), T215F (19.9)**	***M41L (2.8)***, ***A62V (1.4)***, *T69A (3.1)*, ***K70R (3)***, ***F77L (2.5)***, ***Y181C (1.1)***, ***K219E (6.4)***
4	304,000	ABC 3TC LPV/r (07)	AZT D4T DDI EFV LPV/r	M36I (41.9) L63P (100), I93L (100)		K103N (67.7), M184V (37.3)	***M41L (5.7)***
5	13,080	TDF FTC ATV/r (10/07)	AZT 3TC EFV (11/05)	V32I (99.8), M36I (99.4), I47V (99.2), I50L (48), I62V (100), I64V (100), A71V (100), L90M (100)	*M46I (28.3)*	M41L (76.3), K103N (46.9), M184V (21.4), **L210W (14.5)**, **T215Y (15.8)**	***A62V (7.9)***
6	100,000	ABC 3TC EFV (10/07)	AZT ABC DDI IND/r ATV/r (11/00)	M36I (98.4), I64V (99.3)	*I13V (1.1)*	L74V (98.7), K101E (87), Y181C (67.5), M184V (82.2), G190S (72.2)	***K101N (1.9), Y188C (1.9), G190E (4.2), T215Y (4.1), K219Q (4.3)***
7	28,300	AZT 3TC ABC TDF EFV (04)	D4T AMP/r IND/r SQV/r LPV/r	M36I (98.9), L63P (99.5), A71V (98.4), N88D (96.1), L90M (97.6)		M41L (94.1), E44A (99.1), L74I (78.8), K103N (89), V108I (76), V118I (61.6), M184V (98.9), L210W (99.7), T215Y (98.3)	***L74V (3.4)***, *L100I (21.2), K103E (3.4), K219N (25)*
8	29,000	DDI D4T TDF (04)	unknown	I13V (100), L63P (99.8), I64V (100)	*L33I (18.9), M36I (4.9)*	A98G (40)	*K70N (6.9)*, ***V108I (11.9)***, *V118I (30.5)*, ***Y181C (8)***
9	2,600	AZT ABC 3TC (04)	D4T DDI 3TC NVP NFV	M36I (20.1), I64V (86.4)	*V77I (18.2)*	M41L (30.7), M184V (58.9), T215Y (58)	*A98G (23.9), K101E (18.6), V118I (5.6)*, ***Y181C (14.1)***, ***L210W (14.1)***
10	17,100	AZT ABC 3TC (04)	unknown	L63P (97.8), I64L (81.4)	*D60E (0.8)*	K101E (79.8), K103N (79.7), M184V (94)	

Pt.	Viral Load	Failing ARV regimen	Previous ARV exposure	Protease (% abundance by uds)		Reverse Transcriptase (% abundance by uds)	
				Mutations detected by both ss and uds	Mutations detected by uds only	Mutations detected by both ss and uds	Mutations detected by uds only
11	5,400	3TC LPV/r (04)	unknown	K20R (99.9), M36I (99.1), I54V (57.6), D60E (95.6), L63P (99.8), I64V (21.4), V82A (99.9)	*A71V (1.9)*	K103N (70.1), M184V (99.8)	***A62V (3.5)***, ***T215F (14.4)***
12	82,800	AZT ABC LPV/r (04)	unknown	L63P (100), I64L (99.6)	***D30N (3.5)***, *M36I (13.5), I62V (1.5)*, ***N88D (2.9)***		
13	56,000	ABC EFV NFV (04)	unknown	G16E (99.2), I62V (99.8)	*M36I (1.2), V77I (2.6)*	K101P (95), K103S (83.7), M184V (96.6), T215F (76.9)	***K65R (3.7)***, ***K103N (11.2)*** *, V118I (0.9)*, ***T215S (1.3)***, ***T215Y (5.2)***
14	15,000	TDF 3TC fAMP (04)	TDF 3TC EFV	L10I (91.8), M46I (95.6), I50V (35.5), I64V (99.6), A71V (48.4)	*I13V (2.1), I64L (0.9)*, ***N88D (0.9)***	M184V (99.4)	
15	17,100	TDF DDI SQV (04)	D4T 3TC DDI DDC NVP NFV	I13V (99.9), L63P (99.2)	*M36I (1.5), I62V (11.1), I64L (2.8)*	V75M (96.8), F77L (95.8), V118I (94.6), Y188L (93.9)	*T69N (5.5)*, ***T215F (1.1)***
16	7,900	3TC SQV LPV/r (04)	D4T 3TC NVP SQV/r	L63P (97.2), I64L (96.7), V77I (99.4)	*L10I (27.8), Q58E (1), V62I (8.4), I85V (8.5)*	K101E (72), M184V (98.9), G190S (85)	***M41L (13.1)***, ***E44D (11.7)***, ***K103N (8.6)***, ***V108I (1.5)***, *V118I (2.2)*, ***L210W (4.2)***, ***T215Y (10.6)***
17	89,100	TDF 3TC NFV (04)	AZT 3TC ABC DDI DDC NVP		*M36I (5.2)*	K103N (83)	
18	7,600	TDF DDI SQV (04)	D4T 3TC DDC NVP NFV	I13V (100), I62V (46.5), L63P (98)	*L10I (4.7), D60E (0.8), I64L (2.2), V77I (0.8)*	V75M (99.7), F77L (99.7), V118I (92.2), Y188L (77.6)	*T69N (26.2)*, ***T215N (4.3)***
19	100,000	TDF DDI EFV (04)	AZT 3TC ABC DDI NFV	V77I (99.6)			***K103N (2)***
20	87,300	unknown		L10V (97.5), I13V (97.5), L63P (99.8)	*L10I (0.9), M36I (5.1), V77I (1)*		*V118I (1.1), V179D (4.2)*
21	100,000	unknown		M36I (98.7), I62V (99.2)	*L63P (0.8)*	K219Q (48.5)	***M184V (16.7)***
22	6,970	unknown		M36I (99.4)	*L110I (9.7), L33F (10)*	K103N (59.2)	***K103E (20.2)***

**NOTE:** (), % abundance by uds.

AZT, zidovudine; ABC, abacavir; 3TC, lamivudine; FTC, emtricitabine; DDI, didanosine.

Mutations in bold, IAS-USA major mutations in low abundance.

Mutation in *italic*, mutations detected by uds only.

TDF, tenofovir; D4T, stavudine; EFV, efavirenz; LPV, lopinavir; SQV, saquinavir; fAMP, fosamprenavir; NLF, nelfinavir; ATV, atazanavir; r, ritonavir boosting.

### Correlation of low-abundance DRMs with subjects' antiretroviral histories

Current failing antiretroviral regimens were known for 19 subjects, and full antiretroviral histories were known for 14 subjects listed in table 1. None of the 10 subjects with low-abundance TAMs (#1, 3, 4, 5, 6, 11, 13, 15, 16, 18) were on zidovudine/stavudine-containing regimens at time of virologic failure. Longitudinal antiretroviral histories were known for 9 of these subjects, and all had prior zidovudine/stavudine exposure. Subjects 5 and 6 had not been on zidovudine for 2 and 7 years, respectively, yet TAMs were still being detected at low levels in their blood. Low-abundance M184V was detected in only one subject (#21) and was not detected in subjects who went off lamivudine/emtricitabine. M184V was detected in 12 of 13 subjects who were on a failing regimen containing lamivudine/emtricitabine and was predominantly present at levels >70%. NNRTI mutations were detected in both low and high abundance in subjects who were on NNRTI-containing regimens either at time of virologic failure or in the past.

Overall, among the 19 subjects with known antiretroviral regimens at time of virologic failure, low-abundance DRMs correlated with the failing regimens in only 4 subjects (21%); all were NNRTI mutations. By comparison, among the 14 subjects with complete longitudinal antiretroviral histories, low-abundance DRMs correlated with historical antiretroviral use in 11 subjects (79%); most were TAMs (OR 13.75, 95% CI 2.5–74.3, p = 0.0016).

### Impact of low-abundance DRMs on the resistance burden in subjects failing ART

We used the Stanford HIV drug resistance scoring algorithm to evaluate the extent to which additional low-abundance DRMs detected by ultra-deep sequencing added to the resistance burden in each subject. Seventeen of 22 subjects (77%) had additional low-abundance DRMs that increased the subjects' genotypic resistance to at least one antiretroviral, and 11 of 22 subjects (50%) harbored new resistance to at least one antiretroviral. For example, in subject 16 the addition of K103N and V108I detected by ultra-deep sequencing to the G190S detected by both methods changed etravirine from intermediate resistance to high level resistance. In the same sample, the addition of M41L, L210W and T215Y to the M184V detected by both methods implied resistance to multiple NRTIs, in addition to lamivudine.

## Discussion

Our study demonstrates that low-abundance HIV DRMs are commonly unrecognized in antiretroviral-experienced subjects at time of virologic failure. DRMs at levels <20% of viral quasi-species made up 36% of the total number of mutations detected by ultra-deep sequencing; the vast majority were undetected by standard HIV genotyping. The additional low-abundance DRMs increased the subjects' genotypic resistance to at least one antiretroviral drug in 77% of subjects and conferred new resistance to at least one antiretroviral drug in 50% of subjects when evaluated with the Stanford HIV drug resistance algorithm. Our findings were in agreement with a prior study by Palmer and colleagues who reported detection of additional 1 to 10 minority drug-resistant variants from each of 26 treatment-experienced subjects failing ART using single-genome sequencing [24]. Our study using a new ultra-deep sequencing method provides additional evidence that the burden of resistance in treatment-experienced subjects at time of virologic failure is greater than that reflected by standard HIV genotyping.

The concept that low-abundance HIV drug-resistant variants can out-compete wild-type virus in presence of drug pressure is a basic evolutionary tenet. When virologic failure is driven by selection of drug-resistant virus, one would expect that mutant variants conferring resistance to a failing antiretroviral regimen to have a relative fitness advantage and to be detected in relatively high abundance compared to wild-type virus. In addition to the question of clinical relevance of low-abundance resistant virus, other more fundamental questions about low-abundance drug-resistant variants detected at time of virologic failure are a) whether they represent accessory mutations or mutations present in succession that increase the level of resistance to the current failing regimens (in which case they would correlate with the failing antiretroviral drug/s) or b) whether they are remnants of resistant populations from prior failed therapy (in which case they would correlate with historical antiretroviral use). The treatment history correlation suggested different associations for different low-abundance DRMs. We found that TAMs when detected at low levels in subjects failing ART were highly correlated with zidovudine/stavudine from prior exposure and not from the current failing regimens. Some of the subjects harboring low-abundance TAMs had not received zidovudine/stavudine for 2 to 7 years. The finding that drug-resistant variants can persist in the circulation for much longer than previously known because of more sensitive sequencing methods support further research using these technologies to understand the mechanism of resistant viral persistence and evolution in an infected person.

M184V was detected in 12 of 13 subjects who were on a failing regimen containing lamivudine/emtricitabine, and all were detected in relatively high abundance (generally >70%). In contrast to TAMs, low-abundance M184V was not detected when subjects were off lamivudine/emtricitabine. The lack of M184V detection in these subjects may be due to M184V variants that were either below the detection limit of this ultra-deep sequencing, were never present, or were cleared from plasma compartment of these subjects. M184V variants may wane from plasma relatively faster than variants carrying other DRMs, probably due to the fitness cost of M184V possessing virus [25], [26].

NNRTI mutations are known to minimally impact viral fitness, and this was observed in our study, in that the majority of subjects (73%) with high-abundance NNRTI mutations were not on NNRTI-containing regimens at time of virologic failure. By comparison, low-abundance NNRTI mutations were seen equally in subjects who were (4 of 9) or were not (5 of 9) on NNRTI-containing regimens. Presence of low-abundance NNRTI mutations in subjects who were not on NNRTI-containing regimens may indicate longer elapsed time from last NNRTI use compared to subjects with high-abundance NNRTI mutations. They may also represent remnant mutations linked in the same genetic backbone of a more favored mutational profile. Longitudinal studies of patients with well-characterized treatment history who stop NNRTI therapy using quantitative full-length sequencing such as single genome sequencing would be desirable.

Interestingly, low-abundance mutations T215F and Y were detected most commonly in subjects who were not on zidovudine/stavudine-containing regimens. T215F and Y mutations require two nucleotide substitutions (T215_ACC_ to 215F_TCC_ or 215Y_TAC_), which is a probable reason for their later appearance during the course of zidovudine failure [27]. When zidovudine/stavudine drug pressure is removed, T215F/Y-containing viral strains would have to reverse-mutate in two different nucleotide positions to return to wild-type. This could therefore explain their prolonged persistence in infected persons off zidovudine/stavudine therapy. In this study, ultra-deep sequencing was able to detect a single revertant T215N at 4.3% in subject #18, essentially capturing a snapshot of evolving HIV strains that may have once been thymidine analog resistant, and providing historical resistance information that would not otherwise be possible by conventional genotyping.

The finding that the additional low-abundance DRMs were more correlated with historical antiretroviral use than to the failing antiretroviral drugs (OR, 13.73; 95% CI, 2.5–74.3, p = 0.0016) is interesting and needs to be confirmed in larger studies, particularly studies with well-characterized antiretroviral histories and with longitudinal deep sequencing. Although the clinical significance of these unrecognized mutations cannot be ascertained due to the cross-sectional nature of our study, a potential clinical implication here is that conventional genotype might be adequate for a subset of patients with well-characterized antiretroviral treatment histories and known longitudinal HIV genotypes. However this clinical scenario does not represent the majority of patients in clinical practice. New sequencing technologies with better sensitivity such as ultra-deep pyrosequencing method can provide important historical resistance information that help clinicians planning subsequent antiretroviral regimens for highly treatment-experienced patients, especially for those whose antiretroviral treatment histories and longitudinal HIV genotypes are not obtainable. Studies have shown that cross-sectional HIV genotyping consistently underestimates mutation rates compared to longitudinal resistance data [28], [29] and longitudinal resistance mutations predict therapy failure better than cross-sectional resistance mutations [30].

Our study has several limitations. First, the findings from the clinical correlation are preliminary in nature due to the relatively small sample size and inherent biases in any retrospective study. Half of the study samples were from an adherence-focused clinical trial [20] and were subjected to selection bias towards highly non-adhered subjects, which might bias the number of minority drug-resistant variants identified. The failing antiretroviral regimens were known for most patients; however, complete antiretroviral treatment histories were obtainable for only 14 subjects in the study. For most subjects the precise treatment time tables and subsequent clinical follow up were not obtainable due to the IRB restriction on use of data that had already been collected in another clinical study. Therefore larger confirmation studies with well-characterized and longitudinal clinical histories are needed. Second, an issue for all HIV genotyping technologies designed to detect low-copy-number viral variants is that sensitivity is dependent on the number of viral templates provided for cDNA synthesis and clonal amplification [9]. Sampling of viral RNA molecules from an extraction solution that follows Poisson distribution is subjected to the stochastic effects of sampling variation. These effects are lessened when the number of template RNA molecules increases (typically >1000) [31]. Since current HIV RNA extraction techniques may only recover up to 10% of viral genomes from a plasma sample [32], only samples with starting RNA copy numbers ≥10,000 can statistically account for the sampling variation. One can still perform HIV genotyping of low-copy-number samples (#3, 9, 11, 16, 18, 22 and 23); however, the levels of mutations identified represent the proportion of sequenced PCR amplicons containing the mutation and may not represent the actual proportion in a plasma sample. We used 140 µl extraction volume because that was the standard plasma volume used for standard sequencing. To obtain a better representative sampling of HIV variants in low-copy-number samples, larger volume extractions from plasma samples would be required. Lastly, ultra-deep sequencing is highly sensitive but more labor-intensive than population sequencing, and the technology will need to be clinically validated before being considered for clinical use.

Despite above limitations, we can conclude that low-abundance HIV drug-resistant mutations commonly go unrecognized in treatment-experienced persons who experience virologic failure. The majority of the unrecognized resistant mutations are clinically-relevant, and they increased the overall burden of resistance in these subjects. The clinical significance of these unrecognized mutations cannot be ascertained due to the cross-sectional nature of our study; however, we can conclude that the majority of unrecognized resistant mutations correlate with historical antiretroviral use rather than to the failing antiretroviral drugs. A potential clinical implication is that ultra-deep sequencing can provide important historical resistance information that help clinicians planning subsequent antiretroviral regimens for highly treatment-experienced patients, particularly for a significant proportion of patients in clinical practice whom treatment histories and longitudinal HIV genotypes are not available.
